# Dihydromyricetin promotes autophagy and apoptosis through ROS-STAT3 signaling in head and neck squamous cell carcinoma

**DOI:** 10.18632/oncotarget.10836

**Published:** 2016-07-25

**Authors:** Teng-Fei Fan, Tian-Fu Wu, Lin-Lin Bu, Si-Rui Ma, Yi-Cun Li, Liang Mao, Zhi-Jun Sun, Wen-Feng Zhang

**Affiliations:** ^1^ The State Key Laboratory Breeding Base of Basic Science of Stomatology & Key Laboratory of Oral Biomedicine Ministry of Education, Wuhan University, Wuhan, China; ^2^ Department of Oral Maxillofacial-Head Neck Oncology, School and Hospital of Stomatology, Wuhan University, Wuhan, China

**Keywords:** dihydromyricetin, autophagy, apoptosis, reactive oxygen species, head and neck squamous cell carcinoma

## Abstract

Chemotherapy is an effective weapon in the battle against cancer, but numerous cancer patients are either not sensitive to chemotherapy or develop drug resistance to current chemotherapy regimens. Therefore, an effective chemotherapy mechanism that enhances tumor sensitivity to chemotherapeutics is urgently needed. The aim of the present study was to determine the antitumor activity of dihydromyricetin (DHM) on head and neck squamous cell carcinoma (HNSCC) and its underlying mechanisms. We demonstrated that DHM can markedly induce apoptotic cell death and autophagy in HNSCC cells. Meanwhile, increased autophagy inhibited apoptosis. Pharmacological or genetic inhibition of autophagy further sensitized the HNSCC cells to DHM-induced apoptosis. Mechanistic analysis showed that the antitumor of DHM may be due to the activation phosphorylation of signal transducer and activator of transcription 3 (p-STAT3), which contributed to autophagy. Importantly, DHM triggered reactive oxygen species (ROS) generation in the HNSCC cells and the levels of ROS decreased with N-acetyl-cysteine (NAC), a ROS scavenger. Moreover, NAC abrogated the effects of DHM on STAT3-dependent autophagy. Overall, the following critical issues were observed: first, DHM increased the p-STAT3-dependent autophagy by generating ROS-signaling pathways in head and neck squamous cell carcinoma. Second, inhibiting autophagy could enhance DHM-induced apoptosis in head and neck squamous cell carcinoma.

## INTRODUCTION

Head and neck squamous cell carcinoma (HNSCC) is the seventh most common type of cancer worldwide, with more than 600,000 new cases reported each year [1]. Despite advances in multimodality treatment, the survival rate of HNSCC patients has improved minimally and persistent mortality is largely due to high rates of regional metastasis, locoregional recurrence, and drug resistance to current chemotherapy regimens [2, 3]. Therefore, new agents with strong efficacy in HNSCC treatment need to be developed.

A growing number of recent studies have focused on chemopreventive activities of phytochemicals because of their low toxicity and potent efficacy [4–6]. Dihydromyricetin (DHM), a 2,3-dihydroflavonol compound, is the main bioactive component extracted from *Ampelopsis grossedentata*. Recently, DHM has been shown to be effective in fighting certain cancer cells and is therefore a promising drug in anticancer therapy [7–9]. Specifically, one study has revealed that DHM causes cell cycle arrest in human hepatoma cells [10]. However, the underlying mechanism remains obscure. Interestingly, several studies have revealed that DHM could potently stimulate autophagic flux [11, 12]. This finding indicates that autophagy may be a novel mechanism by which cancer cells respond to DHM.

Autophagy, an evolutionarily conserved pathway, plays a crucial role in degrading a wide variety of cellular components [13, 14]. Autophagy can either suppress or promote tumor cell growth in different cellular contexts [15–18]. Although the circumstances under which autophagy functions as a primary mechanism of cell death or survival remain to be defined, a hypothesis is that autophagy inhibition promotes apoptosis in cancer cells with intact apoptotic signaling pathways [19, 20]. However, the exact role and mechanism of autophagy in different cancer types and different stages is crucial to identify new methods for cancer therapy and to improve therapeutic efficiency. Signal transducer and activator of transcription 3 (STAT3) acts as a signal transducer as well as a transcription factor, and plays key roles in apoptosis resistance through which malignant cells evade cell death [21]. Recent studies have suggested a correlation among autophagic markers such as LC3B, p62, Atg5, and active STAT3 in some human cancers [15, 20, 21], among which the cancers with the highest STAT3 expression showed an increased expression of autophagic markers and had the worst outcome [15, 22].

In the present study, we report that DHM induces obvious apoptosis in HNSCC cells. Meanwhile, apoptosis is not the sole consequence of DHM deprivation, as DHM treatment rapidly activates an autophagic process. A link between DHM-induced autophagy and ROS production has also been observed. Pharmacological or genetic inhibition of STAT3-dependent autophagy sensitized DHM-induced apoptosis in HNSCC. These findings indicate that autophagy provides a cytoprotective mechanism in HNSCC cells treated by DHM, and inhibition of autophagy may improve the therapeutic efficacy of DHM in HNSCC treatment.

## RESULTS

### DHM induces apoptosis in HNSCC cells

**Figure 1:**
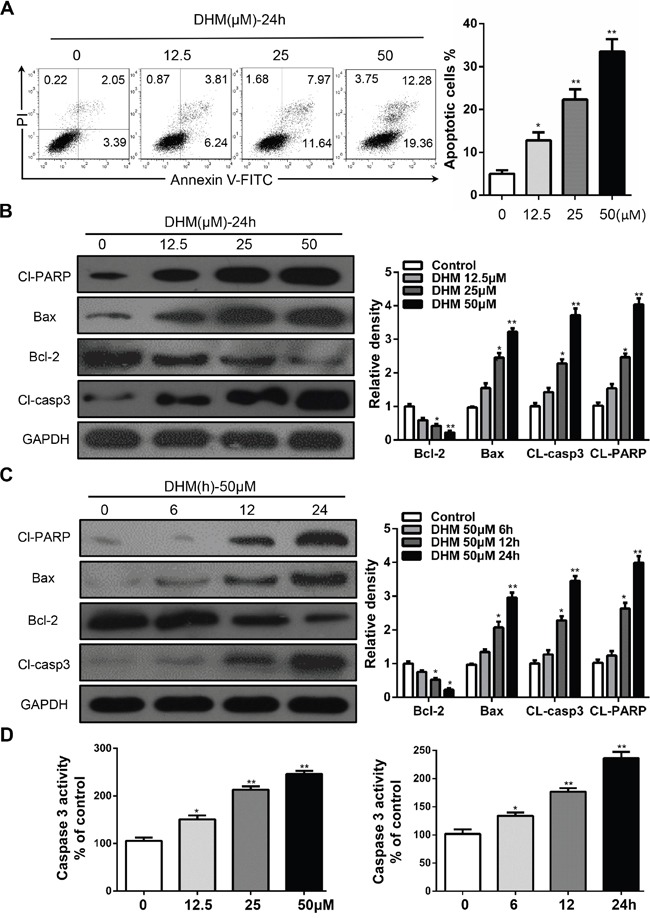
DHM induces apoptosis in HNSCC cells **A.** Cal27 cells were treated with 12.5 μM, 25 μM, and 50 μM of DHM for 24 h and stained with Annexin V/PI, then analyzed by flow cytometry. The percentages of Annexin V-positive cells were presented in bar charts; **B.** Cal27 cells were treated with different concentration of DHM for 24 h then western blot analysis was performed to assess the expression level of cleaved-PARP (Cl-PARP), Bcl-2 and Bax, cleaved-caspase3(Cl-casp3), GAPDH served as a loading control; **C.** Cal27 cells were treated with 50 μM of DHM for 6, 12 and 24 h then western blot analysis was performed to assess the expression level of Cl-PARP, Bcl-2, Cl-casp3 and Bax, GAPDH served as a loading control; **D.** Caspase-3 activity of Cal27 cells after 24 h of DHM treatment at the indicated concentrations. The percentages of Caspase-3 activity were presented in bar charts. The data were presented as the means ± SEM. One-way ANOVA with post-Dunnett analysis was performed using GraphPadPrism 5. *P<0.05, **P<0.01 versus the control group. The experiments were repeated twice.

To determine whether DHM causes cell death by apoptosis, Cal27 cells were analyzed by flow cytometry following Annexin V-FITC and propidium iodide dual labeling. As shown in Figure 1A, cells were treated with different concentrations of DHM for 24 h or 50 μM DHM for 6, 12, and 24 h. We found that DHM induced cell apoptosis in a dose- and time-dependent manner. As key executors of cell apoptosis, both cleaved PARP and the ratio of Bax to Bcl-2 proteins increased in Cal27 cells when treated with DHM for 24 h or with 50 μM DHM for indicated time points (Figure 1B and 1C). We also found that treatment with DHM increased Caspase-3 activity in a dose- and time-dependent manner (Figure 1D). Overall, these results demonstrate that apoptosis was involved in the response of Cal27 cells to DHM treatment. The in vitro inhibition experiment was repeated in another cell line FaDu (Supplementary Figure S1).

### DHM induces autophagy in HNSCC cells

**Figure 2:**
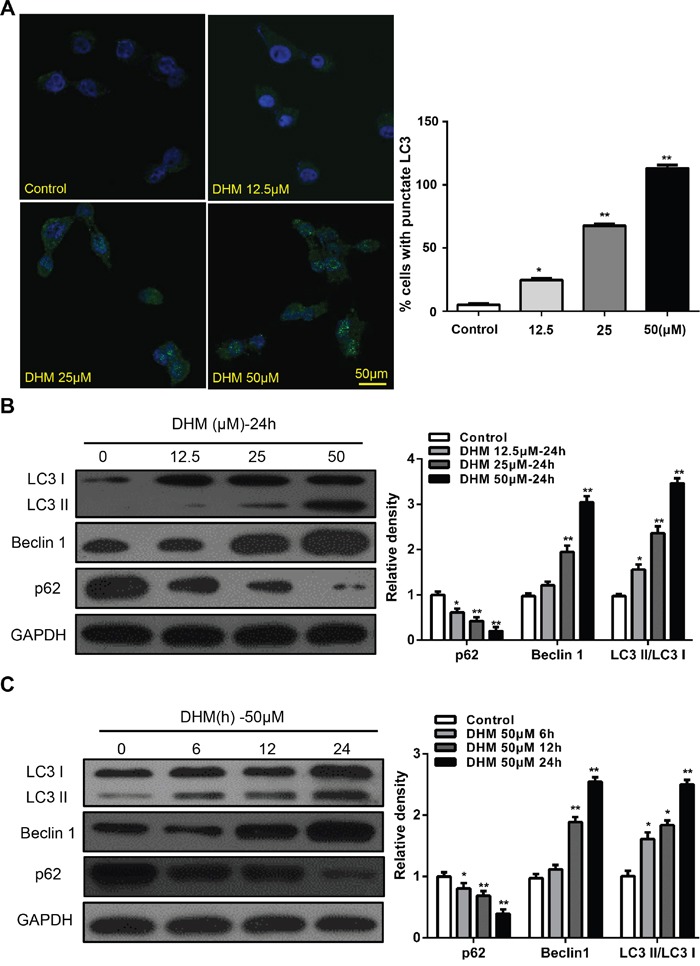
DHM induces autophagy in HNSCC cells **A.** Cal27 cells transfected with GFP-LC3 plasmid were treated with different concentration of DHM for 24 h. The formation of GFP-LC3 puncta were examined using immunofluorescence and quantified. *Scale bar* 50 μm; **B.** Cal27 cells were treated with different concentration of DHM for 24 h, and then the autophagy-associated proteins LC3-I/II, p62, and Beclin1 were detected by western blot analysis. GAPDH served as a loading control; **C.** Cal27 cells were treated with 50 μM of DHM for 6, 12 and 24 h then western blot analysis was performed to assess the expression level of LC3-I/II, p62, and Beclin1, GAPDH served as a loading control; One-way ANOVA with post-Dunett analysis was used by GraphPad Prism5. *P<0.05, **P<0.01 versus the control group. The experiments were repeated twice.

Numerous studies show that autophagy induction is a common event in cancer cells in response to various chemotherapeutic treatments. [23–26] Thus, we examined whether DHM induced autophagy. Microtubule-associated protein light chain 3 (LC3) is a specific marker for autophagy initiation. [20] Autophagosome formation is invariably associated with conversion of LC3 from the cytosolic LC3-I to the autophagosome-associated LC3-II form. [27] We examined whether DHM induced autophagy by LC3. First, we constructed a Cal27 cell line that stably expresses the GFP–LC3 fusion protein and used a fluorescent microscope to detect GFP–LC3 punctate dots [15]. As shown in Figure 2A, after treatment with 50 μM DHM for 24 h, Cal27 cells displayed more green fluorescence than negative controls. Moreover, induction of autophagy was identified by two established measurements of autophagy, that is, enhancement of Beclin-1, a component of class III phosphatidylinositol 3-kinase complex essential for autophagosome formation, and degradation of p62, a protein-facilitating autophagic degradation of ubiquitinated protein aggregation (Figure 2B and 2C). The above experiment was repeated in the FaDu cell line and yielded consistent results (Supplementary Figure S2). These results demonstrated that DHM treatment induced autophagic flux in HNSCC cells.

### Inhibition of DHM-induced autophagy sensitized HNSCC cells to DHM-induced apoptotic cell death

**Figure 3:**
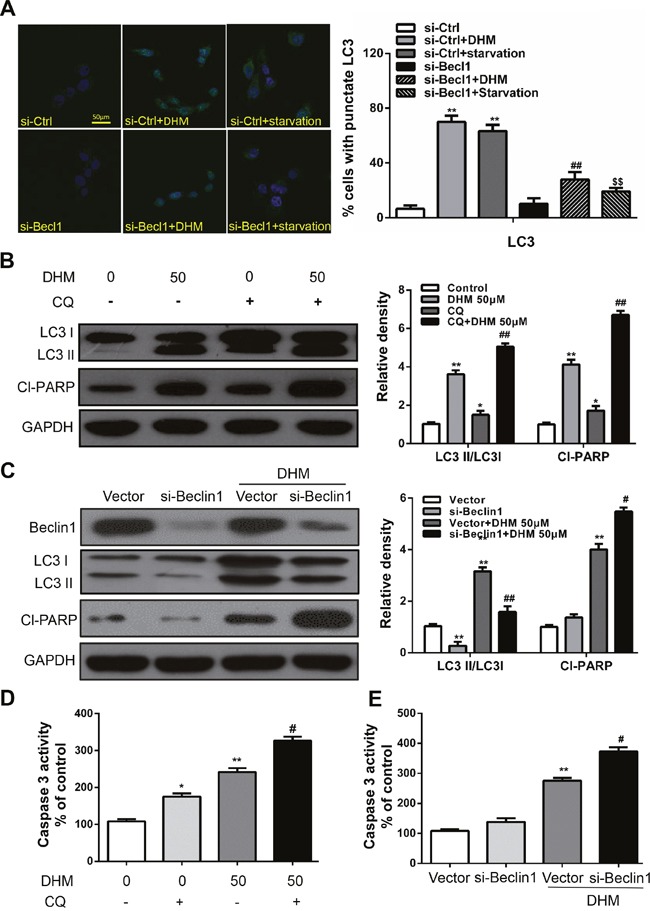
Autophagy alleviates DHM-induced apoptosis in HNSCC cells **A.** Representative images of GFP-LC3 expression patterns in Beclin1 siRNA and control siRNA cells following DHM treatment. Cal27 cells transfected with GFP-LC3 plasmid were used. *Scale bar* 25 μm; Starved cells were used as a positive control for autophagy. One-way ANOVA with post-Dunnett analysis was performed using GraphPad Prism5, **P<0.01 versus the si-control group, ^##^*P*<0.01 versus the si-control+ DHM (50 μM) group, ^SS^*P*<0.01 versus the si-control+starvation group; Cal27 cells were pretreated with 10 μM CQ and incubated with 50 μM DHM for another 24 h. The LC3 and Cl-PARP level **B.** the caspase 3 activity **D.** were determined; The values are presented as the means ± SEM. One-way ANOVA with post-Dunnett analysis was performed using GraphPad Prism5. *P<0.05, **P<0.01 versus the control group, ^#^*P*< 0.05 versus the DHM (50 μM) group; **C.** Cal27 cells were treated with siRNA for *Beclin1* incubated with 50 μM DHM for another 24 h. The LC3 and Cl-PARP level and the caspase 3 activity **E.** were determined. GAPDH was the internal standard for protein loading. The values are presented as the means ± SEM. One-way ANOVA with post-Dunnett analysis was performed using GraphPad Prism5. **P<0.01 versus the si-control group, ^#^*P*< 0.05, ^##^*P*< 0.01 versus the si-control+ DHM (50 μM) group. The experiments were repeated twice.

Autophagy may serve as a pro-survival or pro-death mechanism in different cellular contexts [15–18]. Considering that manipulation of autophagy may improve the efficacy of anticancer therapeutics, we were eager to determine whether the DHM-elicited autophagy in HNSCC favored cell survival or cell death. Beclin1 is an essential protein for autophagy activation; thus, beclin1 deficiency can significantly interrupt autophagy. [27] Therefore, to determine the precise role of autophagy in DHM-exposed HNSCC cells, autophagy inhibition by Beclin1-siRNA transfection was performed. Cal27 cells transfected with Beclin-1 siRNA showed a reduced level of LC3-II accumulation after DHM treatment compared with a scrambled siRNA control, indicating the involvement of Beclin-1 in DHM-mediated autophagy in HNSCC cells. Meanwhile, starved cells that were used as a positive control for autophagy exhibited significant green fluorescence (Figure 3A). To monitor autophagic flux, we also measured the levels of LC3II and cleaved PARP in the absence or presence of CQ, a lysosome inhibitors, could lead to the aggregation of autophagosomes and increase LC3-II level by blocking the fusion of autophagosomes and lysosomes. [28] We found that a CQ challenge increased the level of LC3II and cleaved PARP in Cal27 cells treated with 50 μM DHM (Figure 3B). In agreement with the data derived from pharmacological inhibitors, knockdown of Beclin-1 by siRNA enhanced the cleaved PARP, as assayed by Western blot analysis (Figure 3C), indicating that autophagy is cytoprotective for DHM-induced apoptotic cell death. In addition, DHM remarkably increased Caspase-3 activity and cell death after Beclin-1-siRNA treatment (Figure 3E). Moreover, the Caspase-3 activity results showed that the pretreatment of Cal27 cells with CQ increased the number of apoptotic cells and cell death (Figure 3D). These results reveal that inhibition of DHM-induced autophagy sensitized the HNSCC cells to DHM-induced apoptotic cell death.

### DHM induced autophagy through activating STAT3 pathway in HNSCC cells

**Figure 4:**
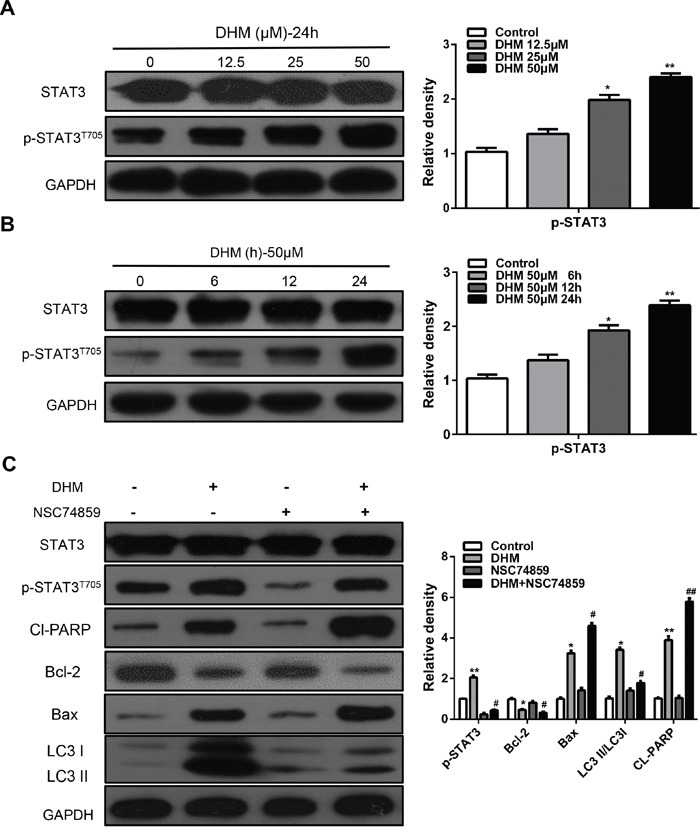
STAT3 signaling pathways are involved in DHM-induced autophagy in HNSCC cells **A.** Cal27 cells were treated with different concentration of DHM for 24 h, the level of STAT3 and p-STAT3^T705^ were analyzed by western blot in left par; **B.** Cal27 cells were treated with 50 μM of DHM for 6, 12 and 24 h, then western blot was performed to analyze the protein STAT3 and p-STAT3^T705^, GAPDH was the internal standard for protein loading. The values are presented as the means ± SEM. One-way ANOVA with post-Dunnett analysis was performed using GraphPad Prism5. *P<0.05, **P<0.01 versus the control group. **C.** CAL27 cells were pretreated with or without 100 μM NSC74859 and incubated with 50 μM DHM for another 24 h. The p-STAT3^T705^, Cl-PARP, Bcl-2, Bax and LC3 level were determined. GAPDH was the internal standard for protein loading. The values are presented as the means ± SEM. One-way ANOVA with post-Dunnett analysis was performed using GraphPad Prism5.*P<0.05, **P<0.01 versus the si-control group, ^#^*P*<0.05, ^##^*P*< 0.01 versus the si-control+ DHM (50 μM) group. The experiments were repeated twice.

STAT3 is a transcription factor that can be activated by IL-6, EGF, and other cytokines, and plays a key role in various biological processes, such as inflammation, cell proliferation, migration, survival, and metabolic disorders [21]. Recent studies have suggested a correlation between autophagy and the activity of STAT3 in human cancer [15, 20, 21]. Thus, we investigated whether STAT3 is involved in DHM action in HNSCC cells. Western blot analysis showed that DHM increased the levels of p-STAT3^T705^ in a dose- and time-dependent manner (Figure 4A and 4B). To further confirm the exact role of STAT3 signaling, we characterized the effects of DHM in cells in which STAT3 was decreased with NSC74859, an inhibitor of the dimerization and phosphorylation of STAT3. After incubation with DHM at an indicated concentration, downregulated STAT3 can inhibit DHM-induced autophagy. The levels of LC3 II/LC3 I was suppressed, indicating that autophagy was increased by activating p-STAT3^T705^ expression. Moreover, Cl-PARP levels increased when p-STAT3^T705^ was suppressed (Figure 4C). These results suggested that DHM induced autophagy through activating STAT3 pathways in HNSCC cells.

### ROS is an upstream signaling molecule that activates the STAT3-dependent autophagy pathway

**Figure 5:**
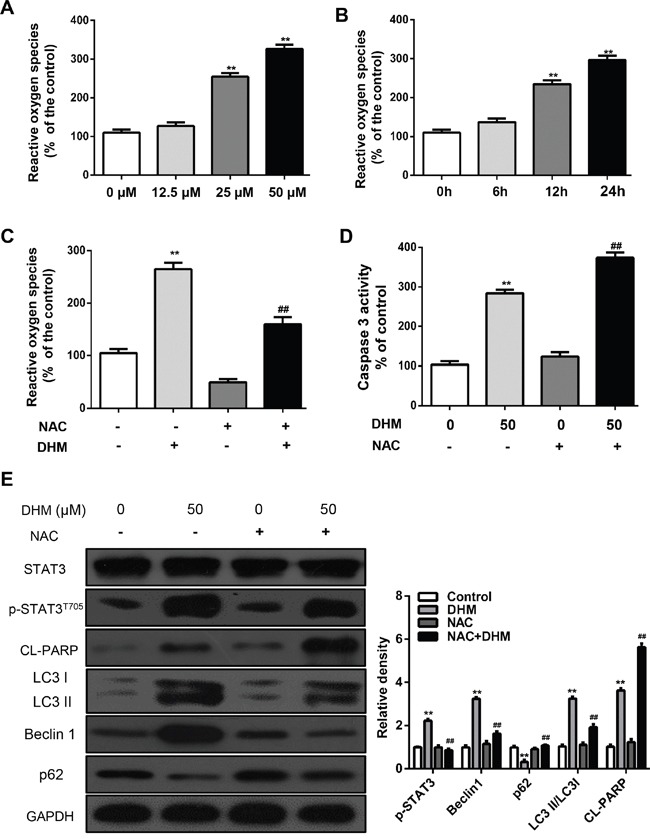
ROS is an upstream signaling molecule that activates the STAT3-autophagy pathway **A.** Cal27 cells were treated with DHM (12.5,25 or 50μM) for 24 h and then examined for the intracellular accumulation of ROS, and the ROS levels were measured using DCFH-DA; **B.** Cal27 cells were treated with DHM (50μM) for 0 h, 6h, 12h and 24h then examined for the intracellular accumulation of ROS, and the ROS levels were measured using DCFH-DA; **C.** Cal27 cells were exposed to NAC (5mM) for 1 h, followed by treatment with DHM (50μM) for 24 h. ROS levels were measured using DCFH-DA. **D.** Cal27 cells were exposed to NAC (5mM) for 1 h, followed by treatment with DHM (50μM) for 24 h, Caspase-3 activity was measured; **E.** Cal27 cells were treated with DHM (50μM) for 24 h after pre incubation with or without NAC (5mM) for 1 h. Levels of p-STAT3^T705^, LC3II/LC3I, Beclin1and p62 were detected by western blot analysis. GAPDH served as a loading control; The values are presented as the means ± SEM. One-way ANOVA with post-Dunnett analysis was performed using GraphPadPrism5.*P<0.05, **P<0.01, versus the control group; ^##^*P*<0.01 versus the DHM (50μM) group. The experiments were repeated twice.

Reactive oxygen species (ROS) is found to be correlated with autophagy [17, 29–31]. We speculate that ROS may be also involved in DHM-mediated autophagy induction in HNSCC cells. To examine this hypothesis, the ROS levels were determined in DHM-treated Cal 27 cells by DCF-DA staining. The elevated ROS levels were proportionally correlated with increasing concentrations of DHM: 50 μM DHM for 12 h or 25 μM for 24 h can significantly generate ROS as revealed by DCFH-DA staining (Figure 5A and 5B). The importance of ROS-STAT in cancer therapy has been reported. A recent study shows Cetuximab and oxaliplatin exhibited antagonistic effects on cellular proliferation and apoptosis through ROS-STAT regulation [32, 33]. So we examined the role of DHM-induced ROS generation in blocking STAT3 signals. In addition, pretreatment with N-acetyl-L-cysteine (NAC), an ROS scavenger [34], reduced the ROS levels induced by DHM (Figure 5C), increased Caspase-3 (Figure 5D), and abrogated DHM-induced apoptosis (Figure 5E). Incubation of cells with NAC for 1 h prior to treatment with DHM revealed that inhibiting ROS generation abrogated the effect of DHM on the phosphorylation of STAT3 (Figure 5D). Moreover, pretreatment with NAC averted the DHM-induced expression of p62, degradation of LC3 II/LC3 I, and enhancement of Beclin 1 (Figure 5D). These results indicated that DHM-induced ROS was an upstream signaling molecule that activates the STAT3-dependent autophagy pathway in HNSCC cells.

## DISCUSSION

Chemotherapy is an effective weapon in the battle against cancer, but numerous cancer patients are either not sensitive to chemotherapy or develop chemotherapy resistance to current chemotherapy regimens, thereby significantly diminishing clinical outcomes [35, 36]. To overcome this challenge, developing several potentially useful therapeutic agents is urgently needed.

**Figure 6:**
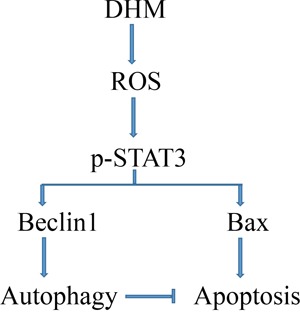
Proposed mechanisms responding to DHM-induced effects in HNSCC

Recent studies have demonstrated that DHM has many biologic effects, including antioxidant [37], anti-tumor [12], and anti-alcohol intoxication activities [38], but the precise molecular mechanisms by which DHM exerts its anticancer effects remain poorly understood. Jin Hong-yong et al. [39] found that DHM induces cell apoptosis through a p53-related pathway in AGS human gastric cancer cells. Xie Jun et al. [40] used a carbon tetrachloride-induced acute liver injury model to demonstrate that DHM alleviated the injury through a JNK-dependent mechanism. Jiang Liang-gui et al. [41] showed that DHM enhanced the chemosensitivity of nedaplatin through regulation of the p53/Bcl-2 pathway in hepatocellular carcinoma cells. However, the results of these basic studies were unsatisfactory because of the problem induced by drug resistance. In the present study, we conducted experiments and determined that the treatment of HNSCC cells with DHM resulted in the induction of cell apoptosis. Furthermore, we investigated the expression of autophagic markers such as LC3, Beclin1, and p62 in HNSCC cells treated with DHM, and we found that autophagic markers were upregulated. Overall, these results suggested that apoptosis was not the sole consequence of DHM deprivation because DHM treatment rapidly activated an autophagic process. We also propose a novel mechanism in which DHM increased the p-STAT3-dependent autophagy by generating ROS-signaling pathways in head and neck squamous cell carcinoma (Figure 6).

Autophagy, an evolutionarily conserved pathway, plays a crucial role in degrading a wide variety of cellular components, such as peroxisomes, the endoplasmic reticulum, and mitochondria [13]. Although drug-induced autophagic tumor cell death has been reported [39, 42], results from most studies support the survival role of autophagy in chemotherapy-induced cell death [15, 20]. Thus, a potential reason for the treatment failure in cancer therapy may be the pro-survival role of autophagy induced by DHM. Thus, we conducted experiments and found that pharmacological or genetic inhibition of autophagy leads to increased DHM-induced apoptosis, thereby indicating that DHM-mediated autophagy was a pro-survival rather than a pro-death mechanism.

To further explore the mechanism of how DHM triggers autophagy, we conducted Western blot to detect the potential proteins involved. STAT3 acts as a signal transducer as well as a transcription factor, and plays key roles in apoptosis resistance through which malignant cells evade death [43]. Recent studies have suggested a correlation among autophagic markers such as LC3B, p62, Atg5, and active STAT3 in some human cancers [15, 20, 21], among which the cancers with the higher STAT3 expression showed increased expression of autophagic markers and exhibited the worst outcome [15, 22]. In the present study, to detect whether STAT3 was involved in DHM-induced autophagy, we evaluated the level of phosphorylated STAT3. We found that DHM treatment led to an increase p-STAT3 level. To further illustrate the role of STAT3 signaling in DHM-induced autophagy, STAT3 was blocked by NSC74859 (also known as S3I-201), an inhibitor of the dimerization and phosphorylation of STAT3 [44]; we found that blocking p-STAT3 could suppress DHM-induced autophagy. Inhibited STAT3-dependent autophagy significantly increased DHM-induced apoptosis.

However, the question remains as to which mechanism mediates the effect of DHM on the enhancement of p-STAT3 expression. Recent evidence suggested that the accumulation of ROS has been linked to multiple pathologies, including neurodegenerative diseases [45], cancer [46], and premature aging [47]. Charlie Mantel et al. [48] demonstrated that phenotyped STAT3−/− mice exhibited mitochondrial dysfunction and increased ROS. Thus, we subsequently examined the correlation between DHM-induced ROS generation and p-STAT3. We found that following the DHM exposure of cancer cells, this agent induced ROS in a time- and dose-dependent manner, an effect that was abolished by pretreatment of cells with NAC. Furthermore, the incubation of HNSCC cells with NAC for 1 h prior to DHM treatment revealed that inhibiting ROS generation abrogated the effect of DHM on p-STAT3. Considering these results, we suggested that ROS was an important cellular mediator that triggers the STAT3-dependent pathway after DHM administration in HNSCC cells.

Thus, we report that DHM induces obvious apoptosis in HNSCC cells. Meanwhile, apoptosis is not the sole consequence of DHM deprivation because DHM treatment rapidly activates an autophagic process. A link between DHM-induced autophagy and ROS production are also observed. Pharmacological or genetic inhibition of STAT3-dependent autophagy sensitizes DHM-induced apoptosis in HNSCC. These findings indicated that autophagy provides a cytoprotective mechanism in HNSCC cells treated by DHM, and inhibiting autophagy may improve the therapeutic efficacy of DHM in treating HNSCC.

## MATERIALS AND METHODS

### Drugs and reagents

Dihydromyricetin, NSC74859 was purchased from Selleck (Houston, TX). N-acetyl cysteine (NAC), Chloroquine (CQ) was obtained from Sigma (St. Louis, MO). Stock solutions were prepared in dimethyl sulfoxide (DMSO), stored at −20°C, and diluted in fresh medium for each experiment. The final concentration of DMSO did not exceed 0.1% in any of the experiments to prevent toxicity to cells. Antibodies against p-STAT3 (T705), STAT3, LC3, and PARP were purchased from Cell Signaling (Beverly, MA, USA); antibodies against Beclin-1, Bcl-2, p62 and Bax were purchased from Santa Cruz (Santa Cruz, CA, USA). Dulbecco's modified Eagle's medium (DMEM) and fetal bovine serum were obtained from Gibco (Life Technologies Gibco/BRL, New York, NY, USA). Beclin1 siRNAs were purchased from Shanghai GenePharma (Shanghai, China). Lipofectamine 2000 was bought from Invitrogen (Carlsbad, CA).

### Cell lines

Human HNSCC cell lines Cal27 and Fadu were bought from the American Type Culture Collection (Manassas, VA). Cells were grown in DMEM (Gibco, Carlsbad, CA) with 10% fetal bovine serum (FBS; Gibco) [49]. All cells were grown in a humidified atmosphere of 95% air 5% CO2 at 37°C and experiments were performed on 4th and 5th passages generated from the frozen stock.

### Apoptosis assay

Apoptosis was quantified with an Annexin V-FITC apoptosis detection kit (BD Biosciences, San Diego, CA, USA) following the manufacturer's instructions as previous described [15].

### GFP-LC3 analysis

Cal27 cells stably expressing GFP-LC3 were obtained by transfecting the cells with EGFP-LC3 plasmid and selected with G418. Transfection using Lipofectamine 2000 Reagent was carried out according to the manufacturer's protocol. After transfection, cells were washed twice with phosphate-buffered saline (PBS), and fresh DMEM was added for further incubation. The images were photographed using a fluorescence microscope (Leica, Brunswick, Germany). The formation of GFP-LC3 punctate structures was examined as previously described [50].

### Caspase-3 activity assay

Caspase-3 activity was determined using a colorimetric assay based on the ability of caspase-3 to change acetyl-Asp-Glu-Val-Asp p-nitroanilide (Ac-DEVD-pNA) into a yellow formazan product (p-nitroanilide (pNA)). An increase in the absorbance at 405 nm was used to quantify the activation of caspase-3 activity. The cells were rinsed with cold PBS and then were lysed with lysis buffer for 15 min on ice. The cell lysates were centrifuged at 18,000 g for 10 min at 4°C. Caspase-3 activity in the supernatant was assayed using the kit (Beyotime, China). Caspase-3 activity was expressed as a percentage of the enzyme activity compared with that of the control.

### Western blot analysis

HNSC cells were treated with the indicated concentrations of DHM pretreated with or without CQ for 24 h. Then the cells were harvested and washed with cold phosphate-buffered saline (PBS). The proteins were extracted with RIPA Cell Lysis Buffer (Beyotime Institute of Biotechnology, Haimen, China), and kept on ice for at least 30 min. The lysates were centrifuged at 12,000 g at 4°C for 10 min, then the supernatant was transferred to a fresh tube. After protein concentration was measured by the bicinchoninic acid (BCA) method, an equal quantity of total protein per lane was separated by sodium dodecyl sulfate-polyacrylamide gel electrophoresis (SDS-PAGE) and transferred to polyvinylidene fluoride (PVDF) membranes. Membranes were blocked with 5% non-fat dry milk and powder in 0.05% Tris-buffered saline and Tween 20 (TBST) for 1h at room temperature, then incubated overnight at 4°C with specialized antibodies. After overnight incubation, membranes were washed for three times and then incubated for 1 h at room temperature with peroxidase-conjugated secondary antibodies. Then blots were developed by West Pico enhanced chemiluminescence detection kit (Thermo) [51].

### Measurement of reactive oxygen species (ROS)

The accumulated cells were treated with dihydromyricetin in the presence or absence of NAC for 2 h and then loaded with 5 mM DCF-DA. After incubation for 30 min at 37°C in a 5% CO2 incubator, the cells were washed twice with HBSS solution and suspended in the complete media, and the fluorescence emission at 525 nm following excitation at 488 nm was measured using a microplate reader. The cellular fluorescence intensity was expressed as the fold change relative to the level observed in the control cells.

### Gene knockdown using siRNA

siRNAs against Beclin-1, as well as control siRNA, were purchased from Shanghai GenePharma. Cells were transfected with siRNA using Lipofectamine 2000 (Invitrogen) according to the manufacturer's instructions [52]. Cells were incubated for 48 h before further treatment.

### Statistical analysis

One-way ANOVA, followed by post-Dunnett, determined the significant differences between the treatment groups. A P value less than 0.05 was used to indicate statistical significance; all of the tests were two-sided, and no corrections were applied for multiple significance testing. All of the experiments were repeated at least three times. Drug and biomarker distributions are represented as the mean values±s.e.m.*, and **, indicate P < 0.05 and P < 0.01, respectively.

## SUPPLEMENTARY DATA AND FIGURES


