# Supplementary material for: Dihydromyricetin promotes autophagy and apoptosis through ROS-STAT3 signaling in head and neck squamous cell carcinoma
Source: Oncotarget. 2016 Jul 25;7(37):59691–703. doi: 10.18632/oncotarget.10836 (PMC5312341; doi:10.18632/oncotarget.10836)
Supplement: Supplementary file 1 [file oncotarget-07-59691-s001.pdf]

Dihydromyricetin promotes autophagy and apoptosis through ROS-STAT3 signaling in head and neck squamous cell carcinoma

SUPPLEMENTARY DATA

Apoptosis assay

Apoptosis was quantified with an Annexin V-FITC apoptosis detection kit (BD Biosciences, San Diego, CA, USA) follow by the manufacturer's instructions as previous described [3]. Briefly, CAL27 cells were exposed to DHM for 24 h, then the cells were harvested and washed with cold PBS, gently re-suspended in Annexin V binding buffer and incubated with Annexin V-FITC/PI. Finally, the cells were analyzed immediately by using a FACS Calibur flow cytometer (Becton-Dickinson, Fullerton, CA, USA).

Western blot analysis

Western blot analysis were performed as previously [1]. Briefly, CAL27 cell lines were treated with the indicated concentrations DHM in DMEM for 24h. Then the cells were lysed, after protein concentration was measured by the bicinchoninic acid (BCA) method, the total protein was separated by using 12% SEMS-polyacrylamide gelelectrophoresis and then transferred onto polyvinylidenefluoride membranes (Millipore, Billerica, MA). Membranes were blocked with 5% non-fat dry milk and powder in 0.05% Tris-buffered saline and Tween 20 (TBST) for 1h at room temperature, then incubated overnight at 4°C with specialized antibodies. After overnight incubation, membranes were washed for three times and then incubated for 1 h at room temperature with peroxidase-conjugated secondary antibodies. Blots were then developed by West Pico enhanced chemiluminescence detection kit (West Pico, Thermo).

REFERENCES

1. Ma SR, Wang WM, Huang CF, Zhang WF, Sun ZJ. Anterior gradient protein 2 expression in high grade head and neck squamous cell carcinoma correlated with cancer stem cell and epithelial mesenchymal transition. Oncotarget. 2015; 6:8807-8821.
2. Wang YF, Zhang W, He KF, Liu B, Zhang L, Zhang WF, Kulkarni AB, Zhao YF, Sun ZJ. Induction of autophagy-dependent cell death by the survivin suppressant YM155 in salivary adenoid cystic carcinoma. Apoptosis. 2014; 19:748-758.
3. Sun ZJ, Chen G, Zhang W, Hu X, Liu Y, Zhou Q, Zhu LX, Zhao YF. Curcumin dually inhibits both mammalian target of rapamycin and nuclear factor-kappaB pathways through a crossed phosphatidylinositol 3-kinase/Akt/IkappaB kinase complex signaling axis in adenoid cystic carcinoma. Mol Pharmacol. 2011; 79:106-118.
4. Sun ZJ, Chen G, Zhang W, Hu X, Huang CF, Wang YF, Jia J, Zhao YF. Mammalian target of rapamycin pathway promotes tumor-induced angiogenesis in adenoid cystic carcinoma: its suppression by isoliquiritigenin through dual activation of c-Jun NH2-terminal kinase and inhibition of extracellular signal-regulated kinase. J Pharmacol Exp Ther. 2010; 334:500-512.

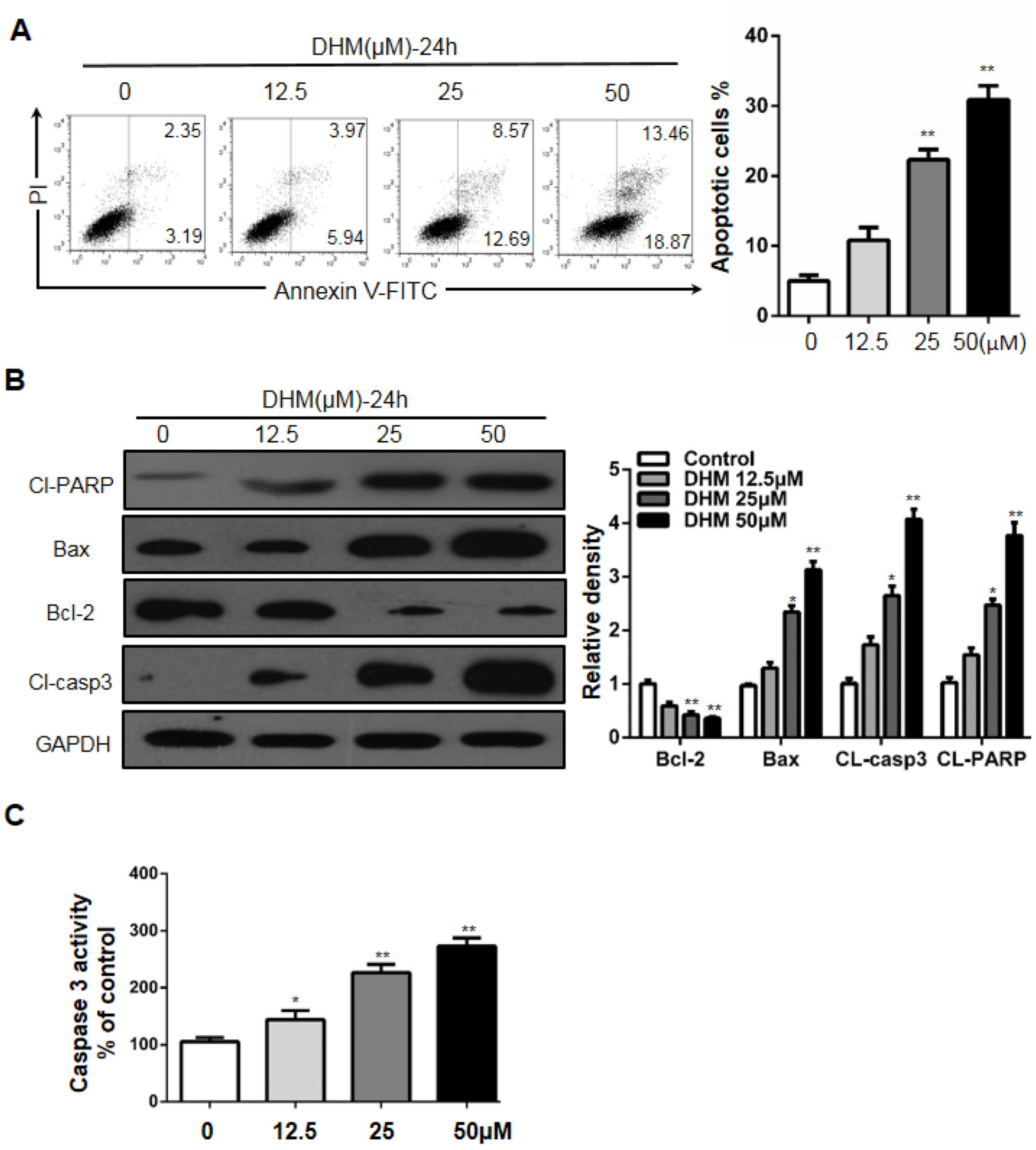

Supplementary Figure S1: DHM induced apoptosis in FaDu cell line. A. FaDu cells were treated with 12.5 μM, 25 μM, 50 μM of DHM for 24 h, and stained with Annexin V/PI, then analyzed by flow cytometry. The percentages of Annexin V-positive cells were presented in bar charts; the data represented mean of three independent experiments. ***P*<0.01, One-way ANOVA with post-Dunett analysis was used by GraphPad Prism5; **B.** FaDu cells were treated with different concentrations of DHM for 24 h then western blot analysis was performed to assess the expression level of cl-PARP, Bax, Bcl-2, cl-casp3 and GAPDH served as a loading control; Relative density data were calculated by Image J, and the data represented mean of three independent experiments. **P*<0.05, ***P*<0.01. **C.** Caspase-3 activity of Fadu cells after 24 h of DHM treatment at the indicated concentrations. The percentages of Caspase-3 activity were presented in bar charts. The data were presented as the means ± SEM. One-way ANOVA with post-Dunnett analysis was performed using GraphPadPrism 5. **P*<0.05, ***P*<0.01 versus the control group.(n=3)

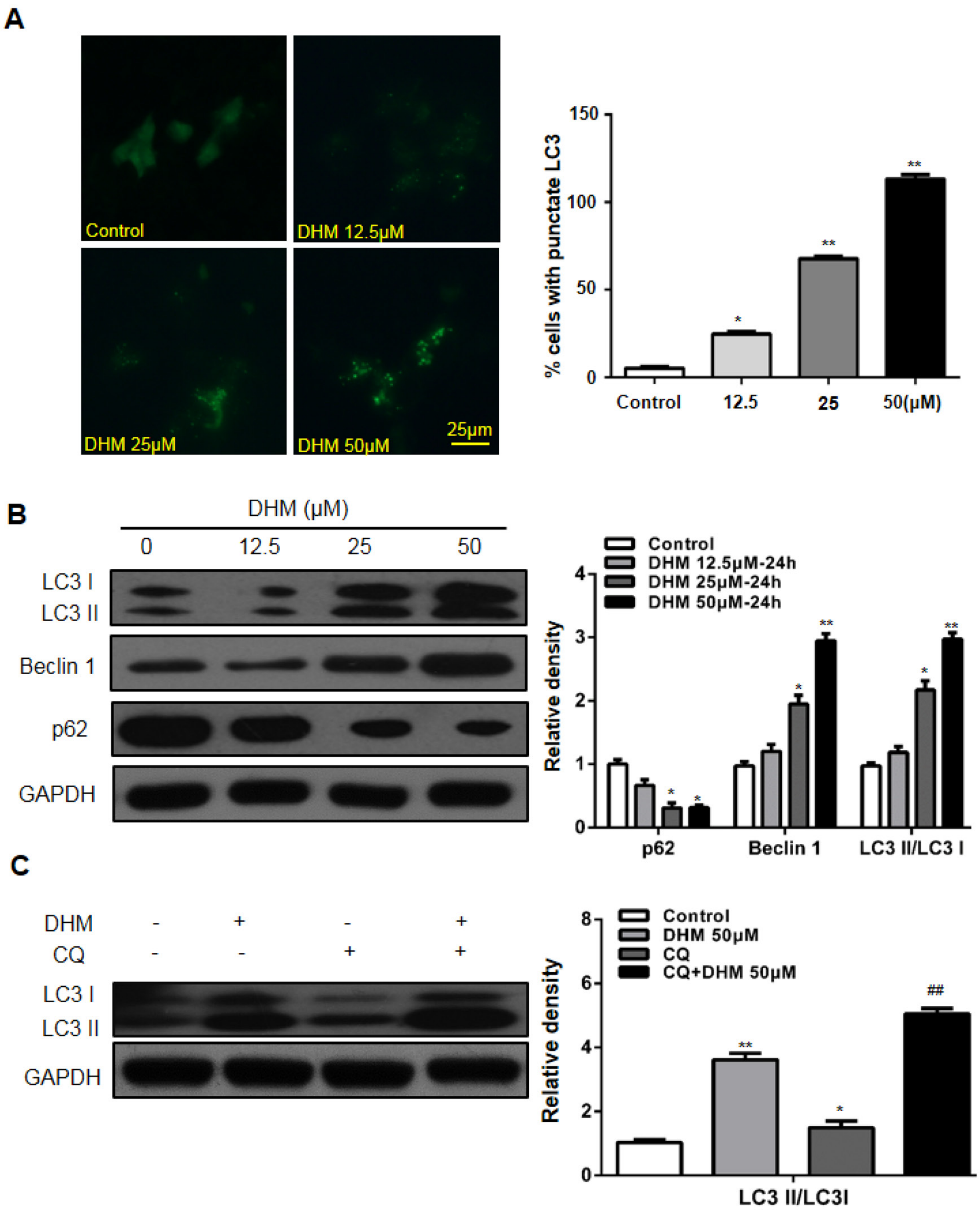

Supplementary Figure S2: DHM induced autophagy in FaDu cell line. A. FaDu cells transfected with GFP-LC3 plasmid were treated with different concentrations of DHM for 24 h. The formation of GFP-LC3 puncta were examined using immunofluorescence and quantified. *Scale bar 25*μm; ***P*<0.01; **B.** FaDu cells were treated with different concentrations of DHM for 24 h, then detected autophagy-associate protein LC3I/II, p62 and Beclin1 by western blot analysis; Densitometric values were quantified using the Image J software, and the data were presented as means ± SEM of three independent experiments. **P*<0.05, ***P*<0.01; **C.** FaDu cells were treated with 50 μM of DHM in the absence or presence of 10 μM CQ, then the expression of LC3II was quantified by normalization of their densitometry to GAPDH; Densitometric values were quantified using the Image J software, and the data were presented as means ± SEM of three independent experiments. **P*<0.05 *versus* the control group, One-way ANOVA with post-Dunett analysis was used by GraphPad Prism5; ##*P*< 0.01 *versus* the DHM (50 μM) group, One-way ANOVA with post-Tukey analysis was used by GraphPad Prism5.
